# Heavy metal pollution and associated health risk assessment of urban dust in Riyadh, Saudi Arabia

**DOI:** 10.1371/journal.pone.0261957

**Published:** 2022-01-06

**Authors:** Abdulaziz G. Alghamdi, Mohamed H. EL-Saeid, Abdulhakim J. Alzahrani, Hesham M. Ibrahim

**Affiliations:** 1 Department of Soil Sciences, College of Food and Agriculture Sciences, King Saud University, Riyadh, Kingdom of Saudi Arabia; 2 Department of Soils and Water, Faculty of Agriculture, Suez Canal University, Ismailia, Egypt; Ghazi University, PAKISTAN

## Abstract

Depending on their particle size and concentration, heavy metals in urban dust pose a health hazard to humans. This study investigated the total concentration, health risk, integrated pollution load index (IPI), and enrichment factor (EF) of various heavy metals in urban dust at different locations in Riyadh City. Surface dust samples were collected from 50 different residential yards in the north, south, west, east, and central corners of the city and analyzed for cadmium (Cd), chromium (Cr), copper (Cu), manganese (Mn), nickel (Ni), lead (Pb), and zinc (Zn). With respect to concentrations heavy metals were in the following order Zn > Cu > Mn > Cr > Ni > Pb > Cd. The EF trends exposed repeated anthropogenic activities were responsible for Mn, Cr, and Ni, while Pb, Zn, and Cu appeared to come from Earth’s crust. Since the heavy metal concentrations were lower than the threshold values, children and adults are exposed to lower health risk in investigated area. Also, there are no pollution of heavy metals in the dust with respect to IPI which is less than the critical limit (<1) with the exception of a sampling location in north side of the city with higher IPI showed unhealthy respiration conditions in particular areas. It was concluded that rapid industrialization and urbanization and their concentrations in dust may cause health problems in near future in north side as well as other sides of Riyadh City.

## Introduction

Dust storms are frequently produced by powerful constant winds acting on loose, fine, and dry soil. The Kingdom of Saudi Arabia is vulnerable to dust storms because of the sparse vegetative cover, drought, light-textured topsoil, and topography [1]. In particular, Riyadh’s capital city is surrounded by desert and is at an altitude of 600 m; thus, it is subjected to numerous dust storms throughout the year. Al-Tayeb and Jarrar [2] estimated that an average of 220-ton km^−2^ year^−1^ of dust falls on Riyadh. Issues associated with dust storms have been highlighted in other countries of Asia, such as China, Japan, and Korea [2]. Chang et al. [3] found that the frequency of emergency visits to National Taiean University Hospital and Shin Guang Memorial Hospital increased 1–3 days after a huge dust storm [3]. Problems were more severe for children aged 0–6 years with illness in the respiratory tract and cardiac patients. Dust adhering to surfaces can lead to significant problems for urban management. Surface dust can easily re-suspend in specific outdoor environments, and adsorbed pollutants may enter the bodies of humans and animals through direct skin contact and respiratory inhalation pathways, which poses a threat to health [4]. Children and the elderly are more susceptible to such toxins because of their immature or age-compromised immune systems [5]. Dust can affect the human body differently depending on characteristics such as the heavy metal concentration, particle size, and particulate matter. Different heavy metals pose different levels of toxicity to the human body [6, 7]. Studies have shown that heavy metals such as Pb can cause the central nervous system to deteriorate by impeding its proper functioning and can persist in children (<6 years and 6–12 years). Pb and Cd have been associated with lower academic and intelligence scores [8]. The Cd is used in a number of industrial processes and introduced into the environment from different point sources (metals manufacture, mining, metal plating, tanneries, battery, pigment and paper industries) leaving aqueous solutions with elevated concentrations [9]. The Zn, Cu, and Cd have also been shown to alter the central nervous system and respiratory system’s roles and may also affect the endocrine system. Pollutants attached to dust may also be transferred to water bodies by runoff, which poses a serious threat to the water quality and human health [10, 11]. Thus, surface dust is a complex environmental medium that acts as a non-point source of pollution [12]. Research has shown that surface dust comprises of inputs from a wide range of sources, such as material from the nearby soils (transported through water), dry and wet atmospheric conditions, particles as a result of paint deterioration, particulate emissions, vehicle fluids, and particles from the weathering of buildings and sidewalks [13]. The size of dust particles has a critical impact on the movement of and it is related to the concentration of contaminants [11]. Heavy metals such as Cu, Pb, Zn, and Cd in surface dust resulting from vehicle emissions, urbanization, and industrial activities are of specific concern because they affect atmospheric and human wellbeing on a large scale [14]. Hence, studying urban surface dust characteristics is important for evaluating the urban environmental quality and its potential effects on human health in the area. Some studies have investigated the heavy metal pollution, distribution, and effects of road dust in several urban areas. However, no data are available on the toxicity and distribution of heavy metals in Riyadh’s surface dust. The primary aim of this study was to identify the particle size distribution of surface dust associated with different areas of the Riyadh city, and to determine the heavy metal concentration in surface dust and calculate the risk to human health. The outcomes of this study will provide significant guidance to Riyadh’s policy makers concerning measures such as replacing vehicles and controlling non-point source pollution to improve the environmental quality and health of children and adults on a long-term basis.

## Materials and methods

### Study site and geography

The investigation was accomplished in Riyadh city, Saudi Arabia. Riyadh city is the capital of Saudi Arabia, with the following geographical points: latitude 24°-08° north and longitude 47°-18° east. Riyadh city is situated above 600 m sea level. It has an area of about 1800 km^2^ and was stated to be populated by approximately 7 million people in the year of 2016 [15]. The investigated area has a very hot summer, with temperatures reaching up to 50°C or more, and an average temperature of 43°C. The overall climate is arid, with very little annual rainfall (22.6 mm); the relative humidity ranges from 10% to 42% throughout the year and winds storms occurred in summer (The General Authority of Meteorology and Environmental Protection (GAMEP), Saudi Arabian Government website: http://www.pme.gov.sa).

### Collection and preparation of dust samples

**Fig 1:**
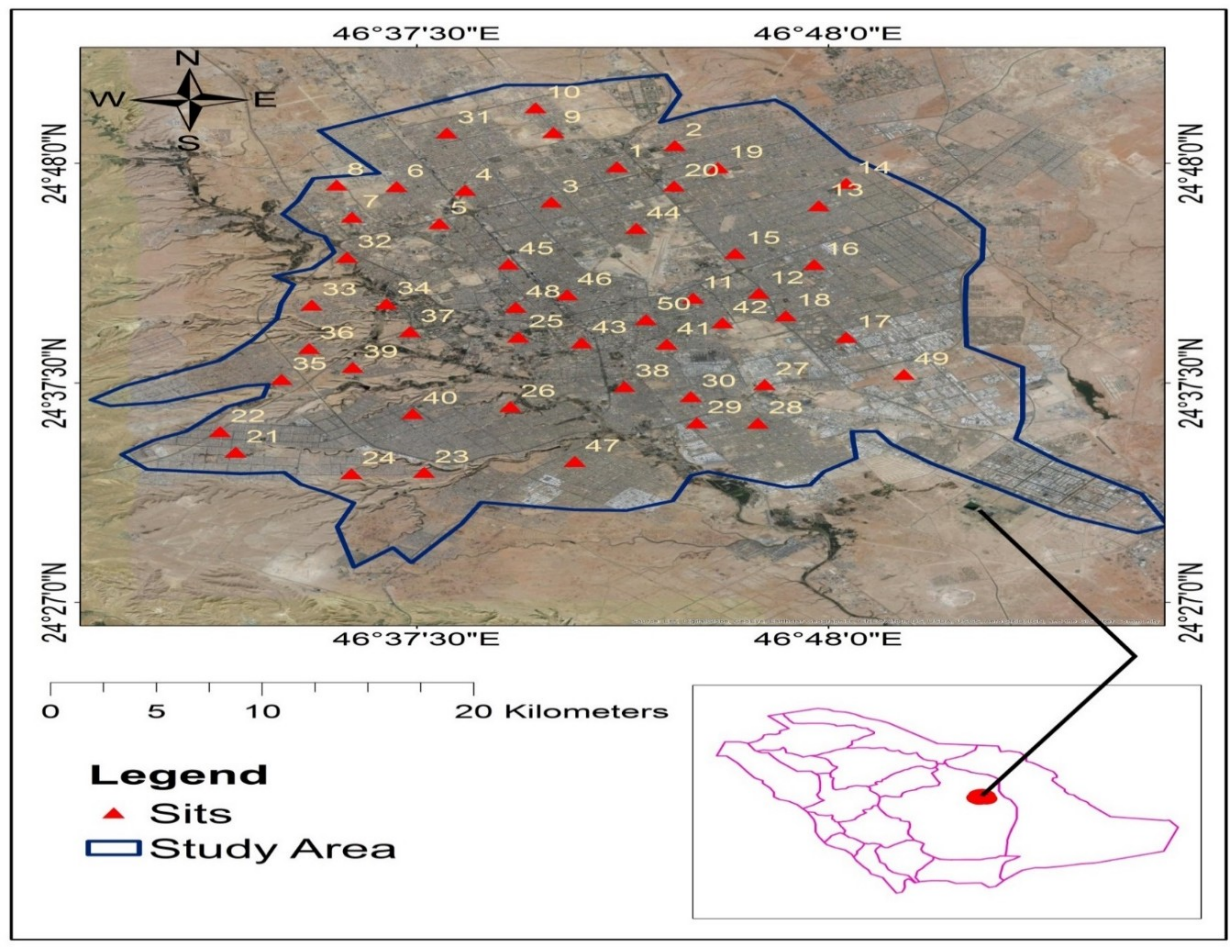
Air dust sampling locations.

Falling dust sampled locations is showed in Fig 1. Samples were obtained from 50 different residential yards in the north, south, west, east, and central areas of the city with a vacuuming method using the Royal Vacuum Brand. The dust samples were taken from the top surface in each area, sealed in plastic bags, and brought back to the Department of Soil Sciences at King Saud University (Riyadh, Saudi Arabia) for analyses. The samples were prepared for measurements by being passed through a sieve with a 100-μm mesh screen to remove building debris and macro biological materials, including rodent droppings [16].

### Analytical techniques

The collected surface dust samples were acid digested (HNO_3_) and analyzed for Cd, Cr, Cu, Mn, Ni, Pb, and Zn. Briefly, 0.5 g of a dust sample was placed in a conical flask and digested with 10 ml of hydrochloric acid on a hot plate. Then, 10 ml of concentrated nitric acid was added. After complete digestion, the remaining solution was filtered and diluted with deionized water. The heavy metal concentrations were analyzed by using inductively coupled plasma–atomic emission spectroscopy (ICP-AES).

### Particle size distribution

A laser diffraction method (Mastersizer 2000, Malvern Instruments Ltd., UK) was used to determine the particle size distribution based on volume and weight. The refractive indices of lactose (1.533) and isopropanol (1.378) were selected, and the default Polydisperse model was used as described [17].

### Statistical analysis

To compare the mean values and outcomes from all parameters, a descriptive statistical analysis technique was performed by using Microsoft Excel ® 2016.

### Quality assurance

The precision, bias, and pollution were assessed by an analytical procedure using reagent blanks and sample replication. The analysis showed that the bias and precision were less than 10%.

### Health risk index

The health risk index is globally applied to measure the potential health risk of surface dust particles [17]. Dermal absorption and hand-to-mouth ingestion have been identified as the major pathways for toxins present in the surface dust to enter the human body. The health risk index involves calculating the following:

$$ADD_{\mathrm{ing}}=C\times BA\frac{\mathrm{Ing}R\times TEF\times ED}{BW\times AT}\times 10^{-6}$$ (1)

$$ADD_{\mathrm{der}}=C\times \frac{SL\times SA\times ABS\times TEF\times ED}{BW\times AT}\times 10^{-6}$$ (2)

*ADD*_*ing*_ is the daily dose from the hand-to-mouth ingestion of substrate particles. *ADD*_*der*_ is the daily dose via dermal absorption of trace elements in particles stuck to exposed skin. Table 1 presents the remaining characteristics and their adopted values. The hazard quotient (HQ) was determined for six heavy metals and involves dividing the daily dose for each exposure pathway by the reference dose (RfD). The hazard index (HI) is the sum of the HQ values. If HQ or HI is less than 1, this indicates no significant risk of non-carcinogenic properties. Suppose HQ or HI is greater than 1, In that case, there is a possibility of non-carcinogenic impacts, and the possibility increases with HQ or HI [18].

**Table 1:** Parameter meaning and value of daily dose model of heavy metals in urban surface dusts.

Parameter	Meaning and Unit	Values		Reference
		Child	Adult	
IngR	ingestion rate, *mg/d*	200	100	[42]
InhR	inhalation rate, m^3^/d	7.6	20	[43]
PEF particle	emission factor, m^3^/kg	1.36×109		[42]
SA exposure	skin area, cm^2^	2800	5700	[42]
SL skin	adherence factor, g/(cm^2^h)	0.2	0.7	[42]
ABS	dermal absorption factor, (unitless)	0.001		[44]
ED	exposure duration, d/y	6	24	[42]
EF	exposure frequency, d/y	180		[44]
BW	average body weight, kg	15	70	[45]
AT	average time, *ds*	ED×365 (for non-carcinogens)		[45]
		70×365 (for carcinogens)		

### Geological accumulation index

The geo-accumulation index is defined as the quantified degree of pollution present in aquatic sediments; it has been extensively used in European trace element experiments [19]. In this study, a modified geo-accumulation index (MI_geo_) was adopted:

$$Ml_{geo}={\mathrm{lg}}^{2}[\frac{c_{\mathrm{sample}}}{1.5\times c_{\mathrm{background}}}]$$ (3)

where C_sample_ is the concentration of a heavy metal in dust and C_background_ is the geochemical background concentration of the heavy metal. MI_geo_ was categorized as described by various scientists [20, 21].

### Pollution load index

The integrated pollution load index (IPI) was calculated by finding the n-root from the n-CFs for all heavy metals [22].


$$IPI=\sqrt[{n}]{CF1\times CF2\times CF3\times \ldots \times CFn}$$ (4)

IPI > 1 indicates pollution, while IPI < 1 indicates no pollution [23].

### Enrichment factor

The enrichment factor (EF) was adopted to distinguish between natural and non-natural background levels of heavy metals in the dust samples [24]:

$$\mathrm{EF}=\frac{{\left(C_{n}/C_{\mathrm{ref}}\right)}_{\mathrm{sample}}}{{\left(B_{n}/B_{\mathrm{ref}}\right)}_{\mathrm{background}}}$$ (5)

Whereas, *Cn* is content of the examined element in the soil, *C*_*ref*_ is content of the examined element in the Earth’s crust, *Bn* is content of the reference element in the soil, and *B*_*ref*_ is content of the reference element in the earth’s crust.

In this study, Fe was selected as the reference element [25]. There is no recognized pollution grade system or classification of the pollution degree based on EF, so a preliminary five-point scale was developed for classification. An EF of less than 2 indicates minimal enrichment, which suggests no or minimal pollution. An EF of 2–5 indicates moderate enrichment, which suggests moderate pollution. An EF of 5–20 indicates significant enrichment, which suggests significant pollution. An EF of 20–40 indicates very high enrichment, which suggests severe pollution. An EF of greater than 40 indicates extreme enrichment, which suggests extreme pollution.

## Results and discussion

### Enrichment factor

**Fig 2:**
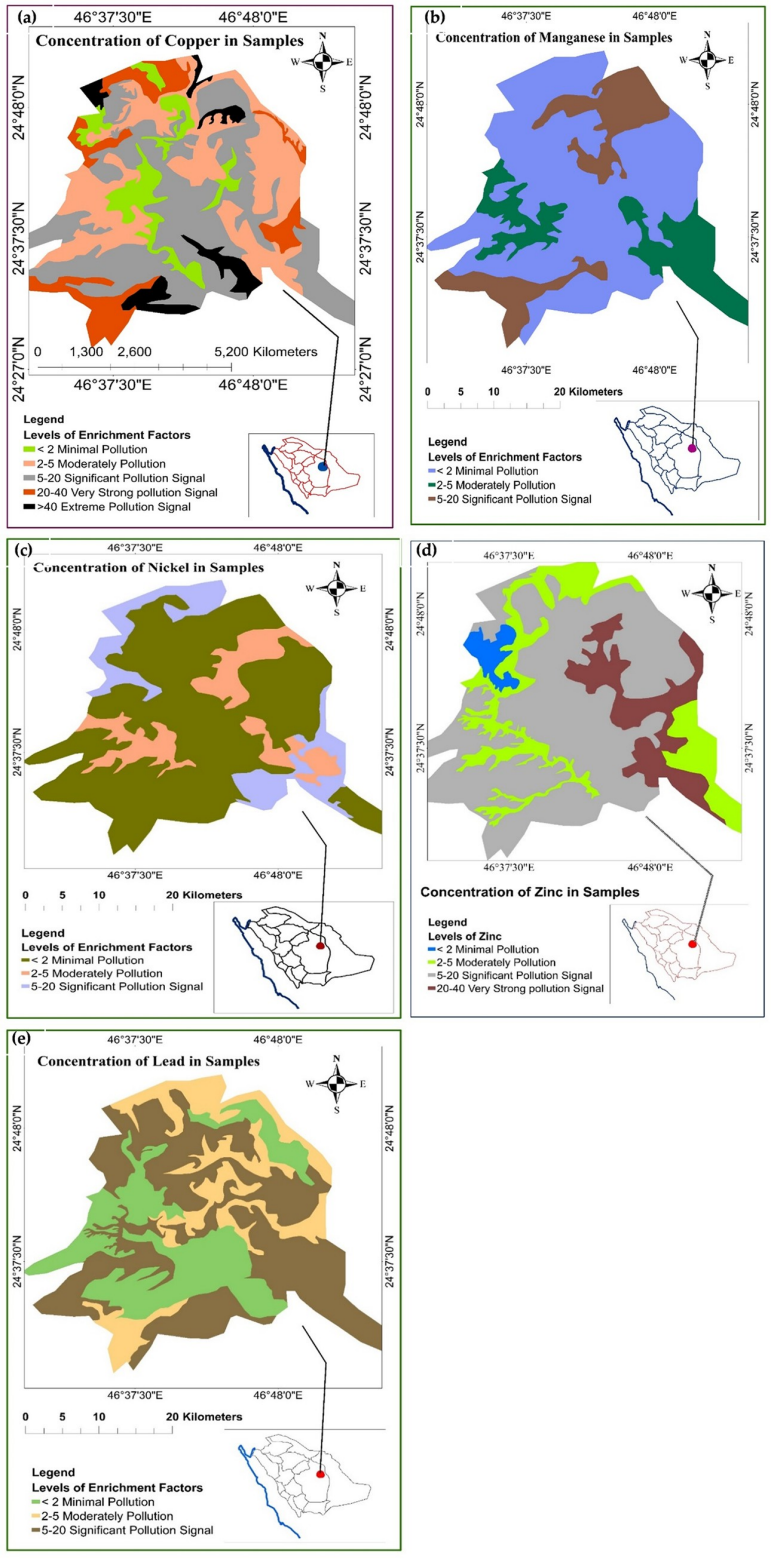
Levels of Enrichment Factors of (a) Copper (b) Manganese (c) Nickel (d) Zinc and (e) Lead in dust samples.

Fig 2(A)–2(E) showed the EF values for five heavy metals based on Eq (5). Two categories of metal enrichment were suggested based on EF values; EF ≤ 2 suggests deficiency to minimal metals enrichment (no pollution) while EF ≥ 2 suggests the high degree of metals enrichment [26]. *EF*_Cr_ was 0.054–1.86 with a mean value of 1.10, which indicates no or minimal pollution of Cr. *EF*_Cu_ was 3.5–108.2 with a mean value of 12.06, which indicates moderate to significant pollution of Cu. *EF*_Mn_ was 0.51–1.53 with a mean value of 0.76, which indicates no pollution of Mn. *EF*_Ni_ was 0.43–2.27 with a mean value of 1.46. *EF*_Pb_ was 0.0–14.8 with a mean value of 6.49. *EF*_Zn_ was 3.3–34.01 with a mean value of 10.87. The EF trend in this study was Mn < Cr < Ni < Pb < Zn < Cu. The spatial variation of *EF*_Zn_ indicated moderate to significant pollution of Zn. *EF*_Pb_ varied for all areas of Riyadh but indicated a significant level of pollution of Pb. Mn, Cr, and Ni had the highest values of EF; this suggests that these elements originated from anthropogenic activities. Meanwhile, Pb, Zn, and Cu may have come from Earth’s crust. Cu, Cd, Pb, and Zn may have multiple sources, while the deposition and accumulation were due to anthropogenic activities such as yellow paint, tires tread, and brake dust. Furthermore, extreme temperatures and the harsh climate may hasten corrosion and cause weathering of wares, lamps, walls, and fences that frequently absorb the heavy metals before they were exposed to urban storms and deposition of dust [27, 28]. Pb was observed even though leaded gasoline was banned in Saudi Arabia in 2001 [29, 30].

### Heavy metal concentration in dust samples

The Cd was below the detection limits of the instruments used in this study. This result agrees with Modaihsh and Mahjou [1], who found no Cd in their study on Granada (a shopping mall located in the eastern ring road highway which is part of Riyadh). The highest Cr concentration (70.28 mg kg^−1^) was in Riyadh’s central area, while the lowest Cr concentration (1.48 mg kg^−1^) was in the south; the mean Cr concentration for all locations was 35.48 mg kg^−1^ [1]. Madany et al. [31] and Al-Rajhi et al. [32] identified Cr and Ni plating and alloys in automobiles and yellow paint on roads as Cr and Ni sources. The area nearby the roads had the highest Cr concentrations. The north area had the highest Cu concentration (1138.11 mg kg^-1^) and the lowest concentration (30.28 mg kg ^1^). The Mn concentration varied among the areas by a significant degree. The highest Mn concentration (212.60 mg kg^−1^) and the lowest Mn concentration (42.22 mg kg^−1^) were observed in the north area. The Ni concentration also varied among the areas with a maximum of 39.73 mg kg^−1^ and minimum of 3.59 mg kg^−1^. Pb was not detected in some areas; the highest Pb concentration (40.13 mg kg^−1^). The highest Zn concentration was 456.05 mg kg^−1^, and the lowest Zn concentration was 36.47 mg kg^−1^. The overall trend for the mean concentrations of heavy metals in dust particles was as follows: Zn > Cu > Mn > Cr > Ni > Pb.

### Particle size analysis

**Fig 3:**
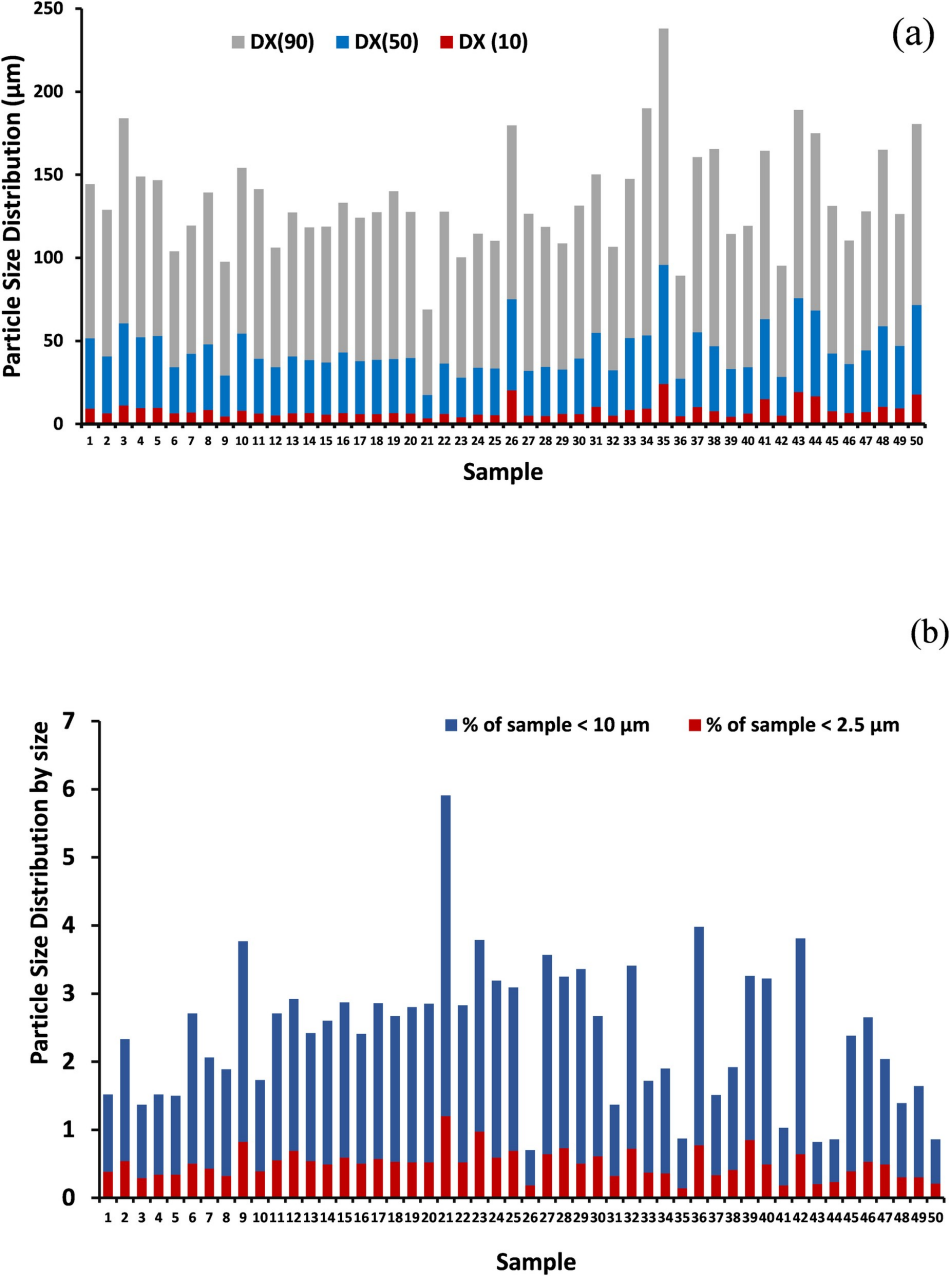
(a) Particle size distribution by volume (b) mass distribution of different size fractions of aerosol PM in the study area.

Fig 3 shows the particle size and volume of the dust samples. Fig 3(A) represents data in D10 50 (median particle size < 10%), indicating the portion of particles with diameters smaller than this value is 10%, DX 50 (median particle size < 50%) indicated the portions of particles with diameters smaller and larger than this value are 50%. The median diameter and the DX 90 (median particle size < 90%) showed the portion of particles with diameters below this value is 90%. The particle size was 3.4–24 μm in DX (10) with a mean value of 8.24 μm, 14–71.8 μm in DX (50) with a mean value of 36.46 μm, and 51.5–142.2 μm in DX (90) with a mean value of 90.61 μm. This gradual increase in particle size was due to large amounts of fine particles suspended in the clouds [33]. Dust poses a hazard to living creatures depending on the particle size and exposure. Some soil properties such as soil texture, electrical conductivity, pH, and organic matter are factors that affect bioavailability of metals and thus influence bio accessibility [34–36]. In this study, the risk assessment was based on the assumption that urban dust and soil have similar particle size distributions [25]. According to the World Health Organization (WHO), an increase in PM10 (i.e., particulate matter with a size of 10 μm or less) of 10 mg m^−3^ annually increases the total mortality by 6%. However, suppose PM10 is increased by 10 mg m^−3^ over several days. In that case, this may cause respiratory tract-related issues such as coughing and other related symptoms. This can result in hospitalization, bronchodilator use, and even death in severe cases [37].

### Health risk index

**Fig 4:**
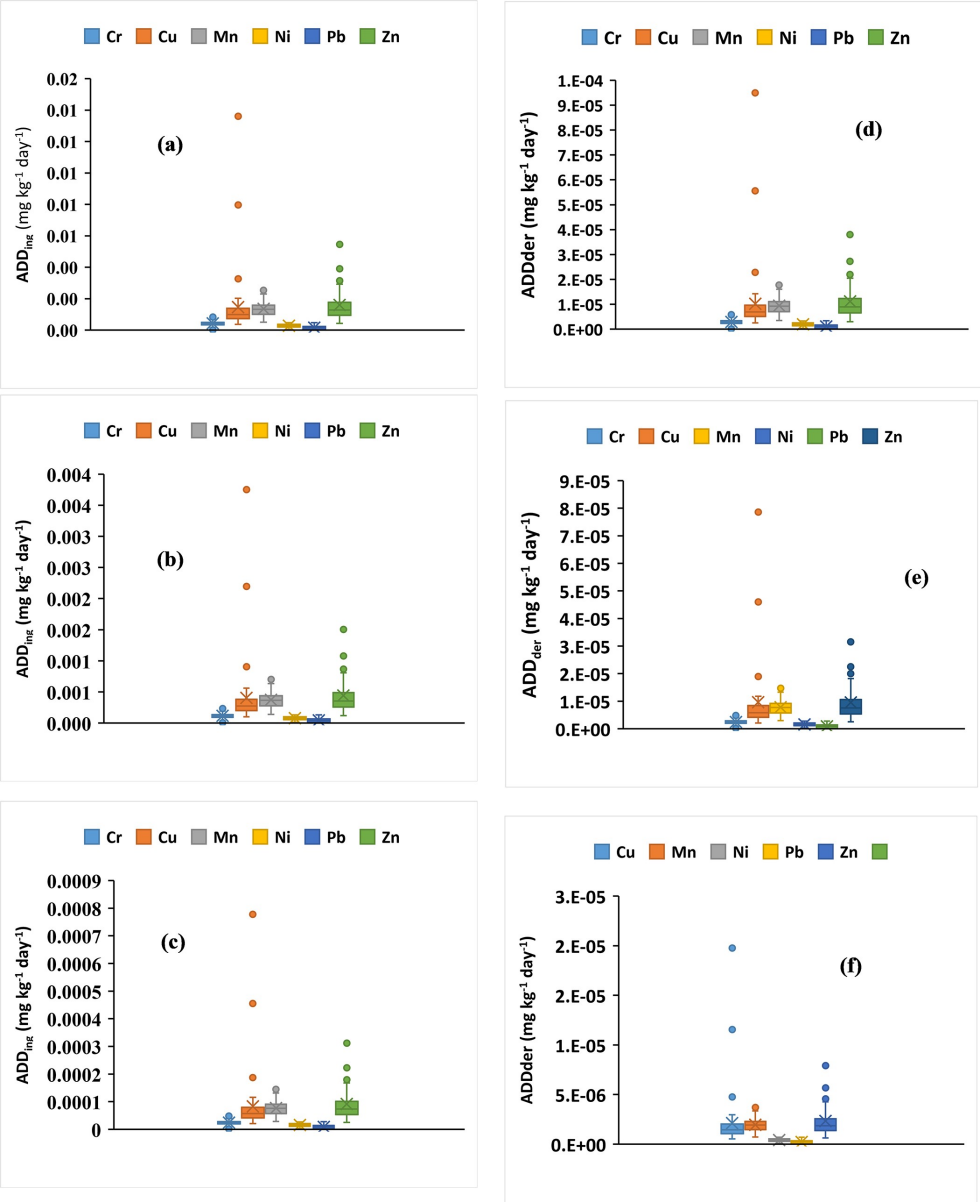
Average daily dose exposure via ingestion in (a) less than 6 years children (b) 6 to 12 years (c) more than 12 years, Average daily dose exposure via dermal in (d) less than 6 years children (e) 6 to 12 years (f) more than 12 years.

Fig 4 presents the ADD_ing_ and ADD_der_ results. The ADD_ing_ could not be calculated for Cd because the Cd concentration was below the detection limit for all collected samples while highest and lowest ADD_ing_ values for Cr were 4.800 × 10^−5^ mg kg^−1^ and 1.0078 × 10^−6^ mg kg^−1^, respectively, for children (<6 years old). The highest and lowest ADD_ing_ values were found in the central and south areas, respectively. The highest and lowest ADD_ing_ values for Cu were 1.36 × 10^−2^ mg kg^−1^ and 3.62 × 10^−4^ mg kg^−1^, respectively, for children (<6 years old). The highest and lowest ADD_ing_ values for Fe were 1.398 × 10^−1^ mg kg^−1^ and 4.99 × 10^−2^ mg kg^−1^, respectively, for children (<6 years old). The highest and lowest ADD_ing_ values for Mn were 2.5 × 10^−3^ mg kg^−1^ and 5.05 × 10^−4^ mg kg^−1^, respectively, for children (<6 years old). Both values for Mn were found in the north area. The highest and lowest ADD_ing_ values for Ni were 4.75 × 10^−4^ mg kg^−1^ and 4.3 × 10^−5^ mg kg^−1^, respectively, for children (<6 years old). The highest and lowest ADD_ing_ values for Pb were 2 × 10^−4^ mg kg^−1^ and 4.8 × 10^−4^ mg kg^−1^, respectively, for children (<6 years old). The highest and lowest ADD_ing_ values for Zn were 5.451 × 10^−3^ mg kg^−1^ and 4.36 × 10^−4^ mg kg^−1^, respectively, for children (<6 years old). Overall, ADD_ing_ values of the determined metals was much higher for children (below six years old) than those who were 6–12 years old and adults (> 12 years). Similar outcomes were obtained [38], who observed exposure to heavy metals from dust in a Zn smelting district. Fang et al. [39] obtained similar results when studying humans’ exposure to heavy metals in surface dust in the Wuhan urban area. The order of ADD_ing_ was Cd < Pb < Ni < Cr < Cu < Mn < Zn for children (<6 years old), Cd < Pb < Ni < Cr < Mn < Cu < Zn for children (6–12 years), and Cd < Pb < Ni < Cr < Mn < Zn < Cu for adults (>12 years). Table 2 indicates that HQ_ing_, HQ_der_, and HI_s_ were lower than 1 for all of the heavy metals. Thus, heavy metal exposure due to surface dust had a relatively light impact on the health of children and adults in Riyadh. The children are more exposed to dust because of their physical activities, including ingesting dust through the mouth, playing and holding toys and other household objects, and licking hands. Therefore, it is important for children to keep good hygiene by ensuring their hands and mouth are always clean and avoid eating without cutlery when they are on the playing grounds like parks and schools.

**Table 2:** Exposure dose, hazard quotient for each element and exposure pathway.

Age	HQ_ing_ (mg/kg dose)					
	Cr	Cu	Mn	Ni	Pb	Zn
0–6	5.88E-03 to 2.80E-01	9.05E-03 to 3.40E-01	3.60E-03 to 1.82E-02	2.15E-03 to 2.37E-02	8.96E-03 to 1.37E-01	1.45E-03 to 1.82E-02
6–12	1.62E-03 to 7.73E-02	2.50E-03 to 9.38E-02	9.94E-04 to 5.01E-03	5.92E-04 to 6.55E-03	2.47E-03 to 3.78E-02	4.01E-04 to 5.01E-03
12+	3.36E-04 to 1.60E-02	5.17E-04 to 1.94E-02	2.06E-04 to 1.04E-03	1.23E-04 to 1.36E-03	5.12E-04 to 7.83E-03	8.30E-05 to 1.04E-03
	**HQ**_**derm**_ **(mg/kg dose)**					
0–6	3.16E-03 to 1.50E-01	6.32E-05 to 2.37E-03	2.52E-05 to 1.27E-04	3.75E-04 to 4.14E-03	6.26E-05 to 9.57E-04	1.01E-05 to 1.27E-04
6–12	2.6E-03 to 1.2E-01	5.2E-05 to 2.0E-03	2.1E-05 to 1.0E-04	3.1E-04 to 3.4E-03	5.2E-05 to 7.9E-04	8.4E-06 to 1.0E-04
12+	6.6E-04 to 3.1E-02	1.3E-05 to 4.9E-04	5.2E-06 to 2.6E-05	7.8E-05 to 8.6E-04	1.3E-05 to 2.0E-04	2.1E-06 to 2.6E-05
	**HI (mg/kg dose)**					
0–6	9.0E-03 to 4.3E-01	9.1E-03 to 3.4E-01	3.6E-03 to 1.8E-02	2.5E-03 to 2.8E-02	9.0E-03 to 1.4E-01	1.5E-03 to 1.8E-02
6–12	4.2E-03 to 2.0E-01	2.5E-03 to 9.6E-02	1.0E-03 to 5.1E-03	9.0E-04 to 1.0E-02	2.5E-03 to 3.9E-02	4.1E-04 to 5.1E-03
12+	9.9E-04 to 4.7E-02	5.3E-04 to 2.0E-02	2.1E-04 to 1.1E-03	2.0E-04 to 2.2E-03	5.3E-04 to 8.0E-03	8.5E-05 to 1.1E-03

### Geological accumulation index

**Fig 5:**
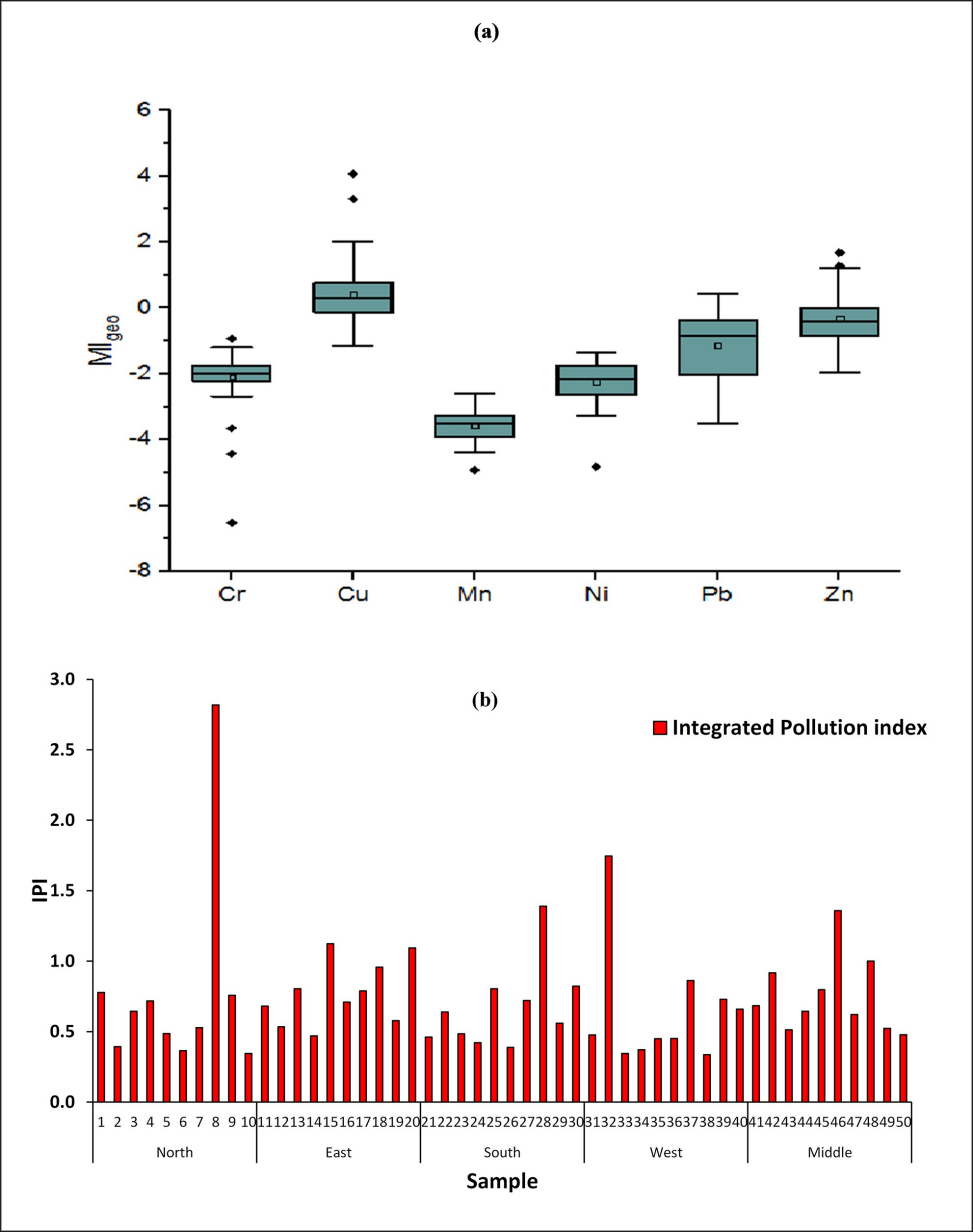
(a) Geological Accumulation Index and (b) Integrated Pollution Index of collected air dust samples in Riyadh City.

Fig 5(A) shows the MI_geo_ results for seven heavy metals in the dust samples. MI_geo_ ranged from -1 to -7 with a mean value of −2 for Cr, from −1 to 4 with a mean value of 0.01 for Cu, from -4 to -3 with a mean value of -3 for Fe, from -5 to -3 with a mean value of −4 for Mn, from -5 to -1 with a mean value of -2 for Ni, from 0 to -4 with a mean value of -1 for Pb, and from −2 to 2 with a mean value of 0 for Zn. Thus, the mean values of MI_geo_ were in the following order: Cu = Zn > Pb > Cr = Ni > Mn. These results indicate that the dust of Riyadh was uncontaminated by Pb, Ni, Fe, and Cr; uncontaminated to moderately contaminated by Zn; and moderately to strongly contaminated by Cu. Shabbaj et al. [40] focused on Jeddah, Saudi Arabia, and found that the road dust was moderate to heavily contaminated with As, Pb, and Zn and heavily to extremely contaminated with Cd. They attributed these results to the increased socioeconomic activities and inadequate disposal procedures for fuel, paint, greases, oil, and used tires, which can increase heavy metal contamination in urban areas [41, 42]. Studies in urban areas have revealed a high mean value of I_geo_ for Pb [43]. In the metropolitan area of Hefei, China, Ali et al. [44] found I_geo_ > 6 for As, which indicates the highest level of pollution.

### Integrated pollution load index

Fig 5(B) shows the IPI results for the heavy metals. Fe was omitted because of its abundance in the soil matrix. About 43 sampling locations had IPI < 1, which indicates no pollution from street dust. However, six locations had IPI > 1 (sample nos. 8, 15, 20, 28, 32, and 46), indicating elevated levels of pollution. Besides, one sample (No. 18) indicated the baseline level of the pollutants. Significantly high IPI values were observed in the east, south, and central areas, and no IPI value was above the limit in the north area. The IPI results indicated that 86% of the sampling locations were not polluted by the accumulation and contamination of heavy metals such as Cr, Cu, Cd, Zn, Mn, Pb, and Ni. These results in accord with those of Alharbi et al. [45], who identified 15 sampling locations where IPI < 1 but also found seven locations where IPI > 1. The east and central areas of Riyadh had the most locations with significantly high values of IPI.

## Conclusion

The present study investigated heavy metals contamination (Cd, Cr, Cu, Mn, Ni, Pb, and Zn) of dust in Riyadh, Saudi Arabia in terms of the health risk index, integrated pollution index, concentration, and spatial variation. The Cd concentration was below the detection limit. About 86% of investigated area were below the threshold level for IPI. For all metals, ADD_ing_ was much higher for children below 6 years old as compared to children who were 6–12 years old and adults (>12 years). Riyadh’s dust was showed uncontaminated by Cr, Cu, Mn, Ni, Pb, and Zn in the light of MI_geo_ results. Particle size analysis showed that many particles were suspended in the air. Higher levels of deposition occurred in the east, south, and central areas of the city, while the deposition level was lower in the north area. Future research should seek to identify the control drivers behind the observed Spatio-temporal trends in dust storm event occurrence across Saudi Arabia, and aim to further explain the significant correlations with human health problems in particular area emerging in last decade.
